# For a healthy start in life, children need smoke-free environments: Progress of the Generation Smoke-Free campaign in Belgium since its launch in 2018

**DOI:** 10.18332/tpc/155920

**Published:** 2023-03-09

**Authors:** Danielle van Kalmthout

**Affiliations:** 1 Belgian Alliance for Smoke Free Society, Brussels, Belgium; 2 The Human Rights Centre, Ghent University, Ghent, Belgium

**Keywords:** smoke-free environments, Smoke-Free Generation, right to grow up smoke-free, Belgium, denormalization of smoking

Belgium still counts 19.4% smokers among its population (15.4% daily smokers)^1^, causing 15000 premature deaths in our country alone, every year^2^. Due to progressive anti-tobacco policy, smoking is now banned in many (indoor) public places.

The Belgian Alliance for a Smoke-Free Society proclaimed its dream on ‘World No Tobacco Day’ of 2018: a society where no one suffers from the consequences of smoking and secondhand smoke. To achieve the first smoke-free generation by 2037, a whole package of mutually reinforcing measures (MPOWER^3^) is needed.

The ‘Generation Smoke-Free’ project aims to ensure that children born in 2019 are protected from tobacco smoke at every stage of their lives and never start to smoke later in life, so that when they come of age in 2037, these children can claim, ‘I belong to the first smoke-free generation’.

Children have the right to grow up smoke-free, which is why the initiators of the Generation Smoke-Free project are fully committed to the *further denormalization of smoking and to increasing the support available to people who want to stop smoking.*

Before looking into the results of the Generation Smoke-Free campaign in Belgium so far, and making some recommendations, this article will first zoom in on some of the arguments used to involve parents and grandparents, municipalities, associations, companies, and others, in making children’s environments smoke-free, and in helping smokers to quit.

## The need for smoke-free outdoor public environments

*Children copy behavior, including smoking behavior*

Even today, hundreds of young people start smoking in Belgium every week. When children see others smoking, it creates the impression that smoking is a normal and enjoyable part of life, rather than a deadly addiction. And children copy behavior: seeing people smoke encourages them to smoke. Children exposed to adults smoking around them are more likely to start smoking, with two-thirds of those experimenting with smoking going on to become regular smokers^4^. But research shows that if smoke-free becomes the norm and there is no smoking in sight, children are less likely to light up a cigarette themselves. A smoke-free environment protects them from tobacco addiction. This strategy is underpinned by the World Health Organization (WHO). The WHO sees the denormalization of smoking in the general population as a key strategy to tackle the tobacco problem among young people. However, smoking is still common in many places frequented by children and young people. This is why Generation Smoke-Free wants to create as many smoke-free environments as possible in areas frequented by children and young people. Smokers are partners to reach this goal and are asked to smoke out of children’s sight.

*Children have the right to grow up in a smoke-free environment*

The right to grow up smoke-free is defended by various articles of the UN Convention on the Rights of the Child (UNCRC), where the right to health (Article 24), the right to life, survival and development (Article 6) are key. In Article 6, the UNCRC goes even further than the right to life and survival as set out in other human rights treaties, because it includes the child’s right to development.

The bodies of children and youngsters are still developing, making them among the most vulnerable members of the population. At least 250 of the 4000 chemicals in tobacco smoke are known to be very harmful to health, and more than 70 of these substances are known to cause cancer^5,6^. Children exposed to passive smoke have a reduced lung function and an increased risk of respiratory infections, asthma, behavioral problems and learning difficulties. Smoking teenagers have less healthy airways and more cardiovascular diseases^7^. Adolescence is also a time when teenagers experiment, including with tobacco, with a high risk of addiction, and an industry keen to find ‘replacement smokers’.

Children and youngsters have the right to grow up healthy and to become healthy adults, without harmful substances adversely affecting their development. Generation Smoke-Free therefore puts children’s rights at the forefront of its campaigns to ensure that present and future generations can grow up smoke-free.

*No more cigarette butts*

Cigarette filters are an environmental hazard and are among the 10 most common plastics in the world’s oceans. Every year, an estimated 4.5 trillion cigarette filters are dumped into the environment^8^.

The main component of cigarette butts is plastic (cellulose acetate). The filter was introduced to reduce the health impact of cigarette smoking. In retrospect, this turned out to be mainly a marketing trick, because research has shown that the filter actually causes smoke to be inhaled more deeply and that fibers from the cellulose acetate may also end up in the lungs. As we now know, the claims that filtered cigarettes were ‘healthier’ were fraudulent^8^. The only thing filters may have done is to make smoking easier and less harsh, increasing both the risk of addiction for smokers and the overall burden of the non-biodegradable and toxic cellulose acetate filters in our environment^9^. Moreover, cigarette butts contain the same toxic chemicals found in cigarettes, such as arsenic and nicotine, threaten our aquatic ecosystems, and are toxic when ingested by children or wildlife^10^.

Smoke-free environments have the additional advantage of reducing the number of cigarette butts that end up on the ground.

## Results so far: from 0 to almost 1000 smoke-free playgrounds in 3 years

Our annual survey sent to Flemish local authorities to assess their awareness of the Generation Smoke-Free project and their activities in the context of the initiative, shows the following results:

- Now, 86% of the municipalities are familiar with Generation Smoke-Free compared to 74% in 2020;
- The majority (57%) of the municipalities indicated that their municipality was actively promoting Generation Smoke-Free in 2021, up from 37% in 2020. Of these 62 municipalities, 41 had launched smoke-free environments by 2021, 11 were in the process of doing so, and 7 had not made any sites smoke-free in 2021 but had done so in the previous year(s); and
- Just over half of the respondents said they would definitely commit to Generation Smoke-Free in 2022.

From all the available information currently at our disposal, we know that a minimum of 951 smoke-free playgrounds and 207 smoke-free sports grounds have been established since 2018. This is an impressive result, knowing that in 2018 we started with 6 pioneer municipalities who made the commitment to smoke-free areas at the time. Some municipalities even went a step further and made other environments smoke-free as well, such as the area around all public entrances to municipal buildings, the area around school gates, etc.

In Wallonia, almost 30 municipalities have started to implement smoke-free environments within the framework of Generation Smoke-Free, so far mainly in the province of Hainaut.

## Some recommendations

*Measure public support*

Before Generation Smoke-Free started to encourage smoke-free outdoor public areas, research was carried out to measure the level of public support for making areas frequented by many children smoke-free.

The smoking survey by the Foundation Against Cancer indicated that 89% of the Belgian population believe that children have the right to grow up smoke-free, and that they support the Generation Smoke-Free principle^11^. Eight out of ten Belgians believe that places frequented by large numbers of children (such as children’s farms, playgrounds, sports facilities, and zoos), as well as hospital campuses, should be smoke-free^12^. Non-smokers and ex-smokers are, according to the survey, more positive about smoke-free environments, and indeed a majority of smokers also back smoking bans in most environments, with the exception of open terraces, train platforms and festivals.

*Create a network of partners to work together towards a first smoke-free generation*

Generation Smoke-Free is a broad movement supported by over 150 local authorities, civil society organizations, sports federations, medical associations, companies, etc. All these partners have signed a Charter endorsing the movement’s mission, vision, goals and approach, and committing to work together towards a first smoke-free generation in Belgium.

Moreover, over 160 municipalities and organizations have obtained the Generation Smoke-Free label by making environments frequented by children smoke-free and by supporting smokers who want to quit. The label can only be obtained if a number of conditions are met: clear signs should indicate that the location is smoke-free, smoking areas are permitted but limited in number and out of sight of children, all stakeholders have been informed that the location is smoke-free and about the new rules, and ashtrays are removed (except in the smoking area, if one exists).

This growing societal support has a positive impact on the political effort to achieve a first smoke-free generation in Belgium by 2037. The more municipalities and organizations support our mission and create smoke-free children’s environments, the greater the support for the political measures that the Alliance for a Smoke-Free Society advocates towards politicians, such as raising taxes, reducing the number of points of sale, or introducing a display-ban.

*Focus on environments frequented by children*

Making outdoor environments frequented by children smoke-free is an important measure to achieve a smoke-free generation. After all, research shows that children will be less inclined to start smoking if it is no longer seen as normal and attractive behaviour^13,14^. The vast majority of smokers (90%) started smoking before the age of 18 years. Experimental behavior is common among adolescents and young people become addicted to nicotine very quickly – much faster than adults. This is because the adolescent brain is much more sensitive to the rewarding effects of nicotine compared to the adult brain. Rewarding stimuli play an important role in the continuation of smoking and the development of nicotine dependence^15^.

The campaign has therefore decided to focus on achieving smoke-free areas in the most significant outdoor spaces where children are present, including care areas, sports grounds, playgrounds, school environments, petting zoos, and youth movements.

Protecting children’s health through the creation of smoke-free environments is central in our communication. A telling example is our annual campaign in the context of the International Children’s Rights Day campaign on 20 November. Here, the right to a smoke-free environment is claimed by children for children.

*An inclusive approach to smokers*

Smokers play a central role in our campaign. After all, making environments smoke-free cannot be done without their help. They are the ones who are being asked to set a good example and to ensure that smoking takes place out of sight of children. In general, smokers are cooperative if the rationale behind the campaign, i.e. protecting children’s health, is explained, because they, too, do not want their children to smoke later on.

More smoke-free environments, including outdoors, make it essential to assist smokers even more in their efforts to stop smoking. The good news is that more than two in three smokers want to quit smoking. But quitting smoking can be difficult. According to the smoking survey of the Foundation Against Cancer, 56% of smokers relapse within 3 months^16^. On average, it takes 5 attempts to stop before they achieve definitive success.

A smoke-free environment invites people who smoke to think about their smoking addiction and therefore provides a very appropriate place to inform smokers how they can get rid of their addiction. Moreover, smoke-free environments also help people who are quitting not to be tempted to start smoking again.

In the framework of our campaign, we have developed specific signposting for any smoking areas located on the premises, out of sight of children, referring to a high quality quitline (Tabakstop), where smokers are given information and are referred, if desired, to professional counselling to help them stop smoking.

## Smoke-free environments: a decisive bottom-up measure

Making outdoor environments smoke-free through positive labelling, encouraging smokers to quit or, if not possible, to smoke out of children’s sight, and teaching children a different norm about smoking, is an important contribution to achieving the first smoke-free generation in Belgium. And this is urgently needed. It is incomprehensible that a consumer product, which kills more than half of its users, is available on every street corner, when it violates human rights, lures countless children worldwide every day into nicotine addiction, causes a tremendous environmental impact, and vastly drives up the costs to be borne by the whole of society.

Smoke-free environments are not the only measure to stop this misery, and their effects will become apparent in the long-term. However, they will ensure that today’s children will stop viewing smoking as a pleasurable experience. They will initiate societal debate on smoking, in sports clubs, schools, hospitals, town councils, zoos, amusement parks and so on. They will make smokers who enter a smoke-free environment reflect on their addiction and perhaps consider seeking professional help to stop. Finally, they will ensure that cigarette butts do not end up as waste in the environment.

The daily achievements of people and communities working together to create smoke-free environments will ensure that the ambition of a smoke-free generation gains a foothold in the whole of society. Such bottom-up support is essential to denormalize smoking and to ensure that children in the future see cigarettes as a curiosity of the past.

## Data Availability

Data sharing is not applicable to this article as no new data were created.
